# Prenatal diagnosis of Williams-Beuren syndrome by ultrasound and chromosomal microarray analysis

**DOI:** 10.1186/s13039-022-00604-2

**Published:** 2022-06-28

**Authors:** Ruibin Huang, Hang Zhou, Fang Fu, Ru Li, Tingying Lei, Yingsi Li, Ken Cheng, You Wang, Xin Yang, Lushan Li, Xiangyi Jing, Yongling Zhang, Fucheng Li, Dongzhi Li, Can Liao

**Affiliations:** 1 Prenatal Diagnostic Center, Guangzhou Women and Children’s Medical Center, Guangzhou Medical University, Guangzhou, Guangdong China; 2 School of Medicine, South China University of Technology, Guangzhou, Guangdong China; 3 Southern Medical University, Guangzhou, Guangdong China

**Keywords:** Williams-Beuren syndrome, Prenatal diagnosis, Chromosomal microarray analysis, Ultrasound

## Abstract

**Background:**

There are a few literature reports of prenatal ultrasound manifestations of Williams-Beuren syndrome. We aimed to explore the prenatal diagnosis of Williams-Beuren syndrome by ultrasound and chromosomal microarray analysis and describe the prenatal ultrasound performance of this syndrome.

**Methods:**

In this retrospective study, we reported eight cases of Williams-Beuren syndrome diagnosed at our prenatal diagnostic center from 2016 to 2021. We systematically reviewed clinical data from these cases, including indications for invasive testing, sonographic findings, QF-PCR results, chromosomal microarray analysis results, and pregnancy outcomes.

**Results:**

In this study, the common ultrasound features were ventricular septal defect (37.5%), intrauterine growth retardation (25%), and aortic coarctation (25%). Moreover, all patients were found to have a common deletion in the Williams-Beuren syndrome chromosome region at the 7q11.23 locus, which contained the elastin gene. Deletion sizes ranged from 1.42 to 2.07 Mb. Seven parents asked for termination of pregnancy, and one patient was lost to follow-up.

**Conclusions:**

This study is the most extensive prenatal study using chromosomal microarray analysis technology for detailed molecular analysis of Williams-Beuren syndrome cases. We reported three cases combined with first-reported ultrasound manifestations. Case 1 was concomitant with multicystic dysplastic kidney and duodenal atresia combined with case 3. Notably, case 4 was combined with multiple cardiovascular malformations: Tetralogy of Fallot, right aortic arch, and supravalvar aortic stenosis. These manifestations expand the intrauterine ultrasound phenotype of Williams-Beuren syndrome in previous literature reports.

## Background

Williams-Beuren syndrome (WBS; OMIM #194050), a multisystem disorder, is caused by a heterozygous microdeletion in the WBS chromosome region (WBSCR) on chromosome 7 at band 7q11.23 [1]. Its incidence is about 1 in 10,000, more common in microdeletion syndrome [2]. There is no apparent family inheritance pattern in WBS. In most cases, it is of sporadic origin, with no predilection for sex or ethnicity; the rare reported cases of familial occurrence suggest an autosomal dominant inheritance pattern [3].

The common deletion/duplication ranges from 1.55 to 1.84 Mb and spans approximately 26–28 genes [4]. Elastin (*ELN*) haploinsufficiency in WBS results in an arteriopathy involving medium- and large-sized arteries leading to lumen narrowing. Furthermore, congenital cardiovascular defects are the most important and common cause of morbidity and death in 80% of patients with WBS [5–7]*.*

WBS has a characteristic constellation of findings. The main clinical manifestations are as follows: facial appearance characteristics, growth retardation, cardiovascular diseases, infantile hypercalcemia, endocrine abnormalities, intellectual disability, and aberrant neurocognitive profile [4]. Typical facial deformities include a small nose, hypoplastic nasal bridge, macrostomia, large and thick lips, prominent cheeks, a small chin, and periorbital fullness [3]. Among endocrine abnormalities associated with WBS, Hypercalcemia is more common. However, there are also relevant literature reports of hypocalcemia [8]. And the content of calcium may be related to the expression of the *BAZ1B* gene. The broad spectrum of clinical manifestations of this disease is related to the size of the deleted fragment and the function of the deleted genes.

About 6% of fetuses with abnormal ultrasonography and normal karyotype can identify clinically significant chromosomal variations through chromosome microarray (CMA) testing [9]. Compared to fluorescence in situ hybridization (FISH) and multiplex ligation-dependent probe amplification (MLPA), CMA has high resolution and high accuracy at the whole genome level. CMA is not only suitable for identifying typical/atypical Copy Number Variations (CNVs) and refining the distal breakpoint for classical or nonclassical WBS but also can identify other potential pathogenic CNVs.

Due to the prenatal ultrasound features of WBS being incomplete and atypical, it is more difficult to diagnose it prenatally. Until now, approximately 22 cases prenatally diagnosed with WBS have been reported in the literature [10–23]. However, because the prognosis of WBS is very abysmal, we need to improve the detection rate and carry out the intrauterine intervention in time. Here we reported on eight other new cases of fetal WBS diagnosed by CMA to delineate the fetal presentation of this syndrome further. As we know, this study is the most extensive prenatal study using CMA technology for detailed molecular analysis of WBS cases.

## Methods

This study was a retrospective one approved by our institutional review board/ethics committee, and informed consent from the patients’ legal representatives was not required. We reviewed consecutive prenatal cases of WBS diagnosed at the Prenatal Diagnosis Center, Guangzhou Women and Children's Medical Center from November 2016 to September 2021. We systematically reviewed clinical data from these cases, including maternal demographics, indications for invasive testing, sonographic findings, QF-PCR results, CMA results, and pregnancy outcomes.

This study included eight singleton pregnancies. Maternal age was 23–38 years, with an average of 30.88 years. At a median gestational age of 28.50 weeks, each patient underwent a routine ultrasound scan. All cases were scanned for fetal anatomy, and associated abnormalities were recorded. After ultrasound screening or genetic counseling, all gravidas underwent an invasive prenatal diagnosis at our hospital. Their initial amniocentesis indications included ultrasound abnormalities (case 1–8), poor fertility history (cases 3, 7, and 8), and advanced maternal age (case 5).

CMA replaced traditional karyotyping as the diagnostic approach for pregnancies with fetal structural anomalies at our center. With the written consent of the parents, amniotic fluid or fetal blood was sampled by amniocentesis or cordocentesis. Quantitative fluorescent polymerase chain reaction (QF-PCR) was used to detect common autosomal aneuploidies and was also used as a tool to detect maternal contamination. The CMA platform used was CytoScan 750 K Array (Affymetrix Inc., Santa Clara, CA, USA), containing 750,436 25-85-mer oligonucleotide probes, including 550,000 nonpolymorphic (NP) probes and 200,436 single nucleotide polymorphic (SNP) probes (0.1 Mb resolution). The process has been described in detail elsewhere [24]. All patients were offered counseling by a maternal–fetal medicine team, including genetic counselors.

## Results

In this study, eight fetuses were diagnosed with WBS by CMA, and we reviewed the intrauterine ultrasound manifestations of these fetuses. Table 1 shows all the clinical features of these eight cases.

**Table 1:** Clinical features in fetuses with Williams-Beuren syndrome

Case number	Maternal age (years)	GA (weeks)	Ultrasound findings	CMA results	Size	Outcome
1	31	23	MCDK	arr7q11.23(72723370–74154209)×1	1.43	TOP
2	27	33	AC	arr7q11.23(72624203–74154497)×1	1.53	TOP
3	29	30	DA	arr7q11.23(72718277–74143060)×1	1.42	Lost to follow-up
4	34	22	TOF, SVAS, RAA	arr7q11.23(72718277–74142190)×1	1.42	TOP
5	38	33	VSD, AC	arr7q11.23(72718278–74143030)×1	1.42	TOP
6	33	31	PAS, IUGR	arr7q11.23(72557180–74628840)×1	2.07	TOP
7	23	27	IUGR	arr7q11.23(72701099–74136633)×1	1.44	TOP
8	32	24	VSD	arr7q11.23(72723371–74141494)×1	1.42	TOP

*GA* gestational age, *CMA* chromosome microarray, *MCDK* multicystic dysplastic kidney, *AC* aortic coarctation, *DA* duodenal atresia, *TOF* Tetralogy of Fallot, *SVAS* supravalvular aortic stenosis, *RAA* right aortic arch, *VSD* ventricular septal defect, *PAS* pulmonary artery stenosis, *IUGR* intrauterine growth retardation, *TOP* termination of pregnancy

**Fig. 1:**
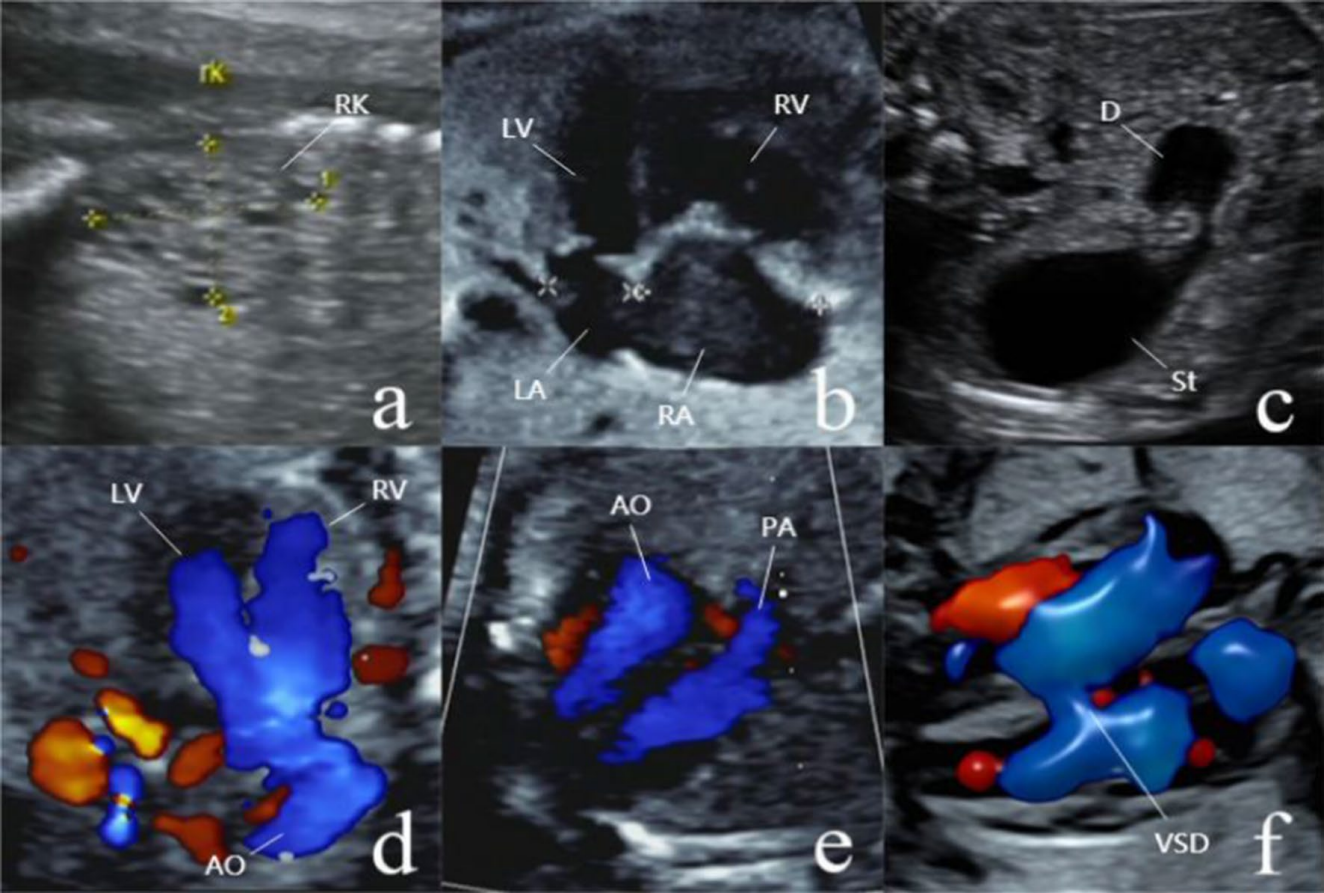
Some typical ultrasound performance in these cases. **a** The right kidney (RK) of Case 1 showed multicystic dysplastic kidney. **b** Case 2 showed atrioventricular ratio is out of balance. **c** The ultrasound image of Case 3 showed the characteristic double bubble sign. **d** The image of Case 4 showed that two blue blood flow signals of the left and right ventricles respectively enter the aorta. **e** Case 5 showed mild stenosis of the pulmonary artery. **f** On the Four-Chamber View, Case 8 showed that the blood flows across the septum. *LV* left ventricle, *LA* left atrium, *RV* right ventricle, *RA* right atrium, *D* duodenum, *St* stomach, *AO* aorta, *PA* pulmonary artery, *VSD* ventricular septal defect

In our eight cases, the common ultrasound features were as follows: ventricular septal defect (VSD) (case 4, 5 and 8, 37.5%), intrauterine growth retardation (IUGR) (case 6 and 7, 25%), aortic coarctation (AC) (case 2 and 5, 25%), multicystic dysplastic kidney (MCDK) (case 1, 12.5%), duodenal atresia (DA) (case 3, 12.5%), pulmonary artery stenosis (PAS) (case 6, 12.5%), Tetralogy of Fallot (TOF) (case 4, 12.5%), right aortic arch (RAA) (case 4, 12.5%), SVAS (case 4, 12.5%). Figure 1 shows some typical ultrasound performance in these cases.

In all cases, the results of QF-PCR suggested that no abnormalities in the number of 21, 18, 13, and sex chromosomes were observed. And in our study, all fetuses were found to have a common deletion in the WBSCR at the 7q11.23 locus, which contained the *ELN* gene. Deletion sizes ranged from 1.42 to 2.07 Mb. This percentage was similar to previous findings.

After genetic counseling on their genomic test results, seven parents asked for termination of pregnancy, and one patient was lost to follow-up.

## Discussion

The current study has preliminarily explored the potential genetic mechanisms of WBS. The deletions in the WBS region arise due to interchromosomal or interchromatid and intrachromatid misalignment resulting in unequal crossing over between the areas comprising the low copy repeats blocks [25]. Our study used CMA as a first-line test and detected eight clinically significant cases. To date, 30 fetuses with prenatal diagnoses of WBS have been reported.

In previous literature reports, the most common ultrasound features were: IUGR (82.35%), SVAS (40%), VSD (30%), AC (20%), and PAS (20%) [10]. However, in our study, the incidence of IUGR (25%) and SVAS (12.5%) were lower than in previous studies. First, IUGR is almost always detected in the late second or third trimester; indeed, the median gestational age at which the diagnosis in our cohort was 28.50 weeks, and 50% (4/8) were in the second trimester, so the intrauterine manifestations of IUGR may not yet be present. Second, this may be due to the improved quality of ultrasound evaluation, and there are some fetal severe defects that can be detected in the first trimester. Moreover, together with the wide application of noninvasive prenatal testing-plus (NIPT-plus), which has high sensitivity and specificity, parents were likely to terminate these pregnancies before a detailed sonographic survey.

As previously reported in the literature, the common intrauterine phenotypes in our cohort were cardiovascular diseases. In addition to the common manifestations such as SVAS, VSD, and PAS, one case (case 4) was combined with TOF, the first-reported ultrasound presentation in a WBS fetus. Similar to previous reports in the literature, the expression of cardiovascular disease in WBS is highly variable, ranging from multiple cardiovascular malformations to no clinical manifestations of this system. Loss of an *ELN* allele is the single most crucial genetic change responsible for the cardiovascular problems of WBS [26]. Due to this reason, decreased vascular elasticity may increase the hemodynamic stress on the endothelium, leading to intimal hyperplasia of smooth muscle and fibroblasts, fibrosis, and narrowing of the vascular lumen [27]. However, the pathogenesis of arterial lesions in WBS may be more complex, and we think there may be other genes that may also be involved in regulating the cardiovascular system. The WBS phenotype may also be affected by the location of genes on both sides of the deletion. Further genetic analysis or epigenetic information is needed to understand the contribution of other genetic components to WBS cognition.

Patients with WBS may have abnormalities of the kidneys or urinary tract. Such as small kidney, renal agenesis, renal insufficiency, renal artery stenosis, bladder diverticula, etc. [4, 28, 29]. Furthermore, MCDK present in case 1 is also a congenital structural defect of the kidney. Among these genes within the commonly deleted interval, no known OMIM disease genes are known to cause urinary defects in fetuses yet. And among the endocrine abnormalities associated with WBS, hypercalcemia is the most common cause. Although various mechanisms have been proposed to cause hypercalcemia, none have been confirmed [30]. We speculate that because of hypercalcemia-induced renal calcium deposits, this may be related to urinary defects, but this requires further experiments to verify.

In addition to the feeding problems and gastroesophageal reflux that occur in infancy, gastrointestinal problems in WBS include colon diverticulosis, inguinal and umbilical hernias, rectal prolapse, constipation, and chronic abdominal pain [31]. Case 3 was combined with DA. The vast majority of cases of DA are sporadic, and it is unclear whether this abnormality is present coincidently or is indeed part of the phenotype of WBS. If the latter is the case, we speculate that it could be because of *ELN* haploinsufficiency, which may lead to the intimal proliferation of smooth muscle and fibroblasts [4]. Hence, more studies are needed to ascertain the precise contribution of ELN or other genes to gastrointestinal problems.

There are some limitations to this study. First, because CMA confirmed the diagnosis of WBS, none of these 8 cases chose to continue with whole-exome sequencing or whole-genome sequencing, and seven decided to terminate the pregnancy to the extent that we cannot discuss it further. Second, we had no information about the CNVs of all patients' parents. Therefore, there was only some genetic information obtained related to CNVs.

In summary, our results suggest that the prenatal ultrasound findings in WBS are mainly characterized by IUGR combined with characteristic cardiovascular abnormalities, but the expression in the cardiovascular system is highly variable, so we recommend CMA for further genetic testing in fetuses with abnormal ultrasound findings.

## Conclusions

In summary, this is the most extensive prenatal study using CMA technology for detailed molecular analysis of WBS cases. The prenatal presentation of WBS is quite variable, but IUGR with cardiovascular complications is the most common ultrasound performance. Although these early sonographic features are non-specific signs, with broad differential diagnosis, they warrant invasive testing. And we reported three cases combined with first-reported intrauterine phenotypes, which expand the intrauterine ultrasound phenotype of WBS in previous literature reports.

## Data Availability

The data that support the findings of this study are not publicly available due to their containing information that could compromise the privacy of research participants. Further inquiries can be directed to the corresponding author.

## Abbreviations

WBS: Williams-Beuren syndrome
WBSCR: WBS chromosome region
ELN: Elastin
SVAS: Supravalvar aortic stenosis
FISH: Fluorescence in situ hybridization
MLPA: Multiplex ligation-dependent probe amplification
CMA: Chromosome microarray
CNVs: Copy number variations
QF-PCR: Quantitative fluorescent polymerase chain reaction
SNP: Single nucleotide polymorphic
VSD: Ventricular septal defect
IUGR: Intrauterine growth retardation
AC: Aortic coarctation
MCDK: Multicystic dysplastic kidney
DA: Duodenal atresia
PAS: Pulmonary artery stenosis
TOF: Tetralogy of Fallot
RAA: Right aortic arch
